# Effect of diode laser on self-assembling peptide enamel remineralization - *In vitro* study

**DOI:** 10.6026/973206300220256

**Published:** 2026-01-31

**Authors:** Pratheek VS

**Affiliations:** 1 Department of Conservative Dentistry and Endodontics, Government Dental College Alappuzha, Vandanam, Kerala, India

**Keywords:** Dental caries, enamel remineralization, self-assembling peptide, P11-4, diode laser, microhardness

## Abstract

The caries lesions of early enamel caries do not have effective non-invasive treatment modalities and thus it is necessary to research on whether the adjunctive diode laser therapy can increase the ability of self-assembled peptide (SAP) biomaterials in remineralization. Eighty premolars had been extracted and randomly separated into four groups (n= 20 each): the control group (artificial saliva), Group B (SAP), Group C (diode laser), and Group D (SAP + diode laser). The process of remineralization lasted 7 days, and the microhardness of surfaces was tested. SAP (P11-4) was very effective on remineralizing the demineralized enamel than the control, whereas diode laser was relatively ineffective. The diode laser used with SAP had synergies with high enamel microhardness recovery. Thus, we show that the use of diode laser can be an effective adjunct in management of early caries through the use of biomimetic remineralizing agents in non- invasive.

## Background:

Dental caries is also one of the most dangerous chronic conditions globally as it affects people of all ages. The interplay between demineralization and remineralization of enamel is dynamic and is mostly due to microbial biofilms, dietary sugars and salivary factors 1]. Traditional restorative therapies though effective tend to compromise good tooth structure and fail to resolve the disease process [2]. Preventive approaches and minimally invasive approaches have become increasingly popular in recent decades, with a focus on facilitating natural remineralization. Self-assembling peptide P11-4 is an innovative biomimetic material that can diffuse in subsurface lesions of the enamel to form three-dimensional matrix that leads to nucleation of hydroxyapatite crystals and repair of the lesions [3]. P11-4 has demonstrated promising results on clinical trials to arrest and reverse early carious lesions [4]. In the meantime, diode lasers and lasers have been explored as caries adjuncts. Laser irradiation changes the crystallinity and permeability of enamel and this may decrease its solubility to acids [5]. Studies show that treatment with the diode laser, particularly in the presence of fluoride or other remineralizing agents, can increase the hardness and resistance of enamel [6]. Nevertheless, there is limited evidence on its impact on SAP treated enamel. Therefore, the current research was carried out to measure and compare the action of self-assembled peptide P11-4, diode laser, and a combination of both on the remineralization process of the artificially demineralized enamel measured through the Vickers microhardness test. The hardest tissue of the human body, enamel has no self-repair ability once it has been demineralized more than a specific depth as it is not cellular and avascular. Nevertheless, during the initial phases of the caries, when the demineralization in the sub surface occurs under an intact surface layer, the lesion may be corrected through natural remineralization caused by the deposition of ions in saliva and exogenous agents [7, 8]. Non-invasive caries management is based on this principle, and its main goals are to establish the mineral balance and maintain the integrity of the tooth structure.

The development of biomimetic substances such as P11-4, marks a new phenomenon in the field of preventative dentistry. P11-4 is self- assembled into 2-sheet fibrils, unlike conventional remineralizing agents which depend on passive diffusion of ions, that is, the lesion body contains a 2-sheet fibrils scaffold, which mimics the natural extracellular matrix [9]. This matrix can serve as a template in hydroxyapatite nucleation and allows deep remineralization of even the lesion subsurface. Both clinical and in situ studies have indicated a profound augmentation in hardness of enamel and deepness of lesions after the utilization of P11-4, indicating its possible use as a regenerative therapy of caries [10]. Conversely, diode lasers which normally have wavelengths in the range of 810-980 nm have been investigated to have potential of thermally modifying the structure of the enamel. Enamel hydroxyapatite crystals melt and recrystallize under laser irradiation resulting in a lower carbonate concentration and lower acid solubility [11]. Moreover, ion diffusion and crystal reorganisation have been demonstrated to increase with the joint application of low-level laser therapy (LLLT) with fluoride, casein phosphopeptide amorphous calcium phosphate (CPP-ACP), and other remineralising agents [12]. It is a dual effect of chemical and structural, which places diode lasers as promising adjuncts in enamel reinforcement. Although the effectiveness of P11-4 and diode laser is individual on enamel remineralization, there is a lack of studies which evaluated the combined effect of these two. In theory, preroxidation of enamel using diode laser can also alter the microstructure of the enamel to increase peptide penetration and overall scaffold formation. On the other hand, peptide laser irradiation that occurs after peptide deposition may favor a greater deposition of the mineral and enhanced crystal incorporation into the peptide backbone [13]. The best clinical protocols involving a combination of biomimetic and photonic technologies in the regeneration of enamel is essential to investigate this synergistic potential. Thus, it is of significance to draw a comparative analysis of remineralization capabilities of self-assembling peptide P11-4, diode laser irradiation, and the combination of both on artificially demineralized enamel. Vickers microhardness testing is a well-known quantitative technique used to monitor the level of recovery of the mineral; the same test is correlated with the strength of enamel. It is hoped that the findings will provide evidence- based suggestions on the least invasive treatment of incipient carious lesions, which combines new biomimetic and laser-assisted techniques. Therefore, it is of interest to comparatively evaluate the remineralization efficacy of self-assembling peptide P11-4, diode laser irradiation, and their combination on artificially demineralized enamel using Vickers microhardness testing, to establish evidence-based protocols for minimally invasive caries management.

## Materials and Methods:

## Study design and setting:

This experimental *in vitro* study was conducted in the Department of Conservative Dentistry and Endodontics, Government Dental College, Trivandrum.

## Sample size and selection:

A total of 80 extracted human premolars, free from caries, cracks, hypoplasia, restorations, or stains, and extracted for orthodontic purposes, were included. Teeth with developmental anomalies, immature apices, or surface defects were excluded.

Sample size (n=20 per group) was calculated based on expected mean difference and standard deviation of microhardness values, with α=0.05 and power=80%.

## Preparation of samples:

Teeth were cleaned and stored in 10% formalin until use. A 5 x 5 mm enamel window was created on the buccal surface by coating the surrounding area with nail varnish. Samples were demineralized using a laboratory-prepared acetic acid solution for 48 hours. Baseline demineralization was confirmed by surface microhardness reduction.

## Group allocation:

Samples were randomly assigned into four groups (n=20 each):

[1] Group A (Control): Stored in artificial saliva.

[2] Group B (SAP): Application of self-assembling peptide P11-4 (Curodont Protect).

[3] Group C (Laser): Diode laser irradiation (960 nm, 2 W, 15 s continuous mode).

[4] Group D (SAP + Laser): SAP application followed by diode laser irradiation.

## Remineralization protocol:

SAP was applied to enamel surfaces with a microbrush and left undisturbed for 2 minutes. For laser groups, irradiation was performed using diode laser. The procedure was repeated daily for 7 days, after which samples were stored in artificial saliva (pH 7.2) between applications.

## Outcome assessment:

Surface microhardness was measured using Vickers microhardness testing machine (Matsuzawa VMT-X7, Japan) with a 100 g load for 15 s. Three indentations per sample were made, and mean Vickers hardness number (VHN) was recorded.

## Statistical analysis:

Data were analyzed using SPSS v18. Descriptive statistics (mean ±SD) were calculated. Intergroup comparisons were performed using one-way ANOVA followed by Tukey's post hoc test. Significance was set at p<0.05.

## Results:

Initial microhardness of sound enamel samples averaged 314.5 ±24.2 VHN. After demineralization, mean hardness reduced significantly to 189.7 ±15.6 VHN, confirming successful lesion creation. Group-wise post-treatment hardness values are presented in Table 1. One-way ANOVA revealed significant differences among groups (F=1523.8, p<0.001). Post hoc Tukey analysis showed Group D significantly higher than Groups A, B, and C (p<0.001), Group B significantly higher than Group C and Group A (p<0.01) & Group C slightly higher than control but not statistically significant (p>0.05) (Table 2). Compared to demineralized baseline, percentage recovery was highest in Group D (57.5%), followed by Group B (38.6%), Group C (6.3%), and Group A (2.1%) (Table 3).

## Discussion:

The *in vitro* experiment showed that self-assembling peptide P11-4 combined with diode laser was the most effective, and then, SAP alone, with the least effects having the diode laser alone. These results indicate a complementary effect of biomimetic peptide-stimulated remineralization and laser-enhanced enamel alteration, which provides a new treatment option in the repair of early enamel lesions. It has been demonstrated that self-assembling peptide P11-4 can be used to create a biomimetic scaffold, which enables deposition and nucleation of hydroxyapatite [14]. The peptide will spontaneously assemble into 2 - sheet fibrils in physiological conditions to form a three-dimensional structure that fakes the natural extracellular matrix of enamel. This scaffold offers points of nucleation of calcium and phosphate ions, which enhance extensive remineralisation in subsurface lesions. The clinical studies have shown that P11-4 has a great regressive effect on the white spot lesions and enhances the enamel surface integrity [15]. These reports are supported by the current findings where SAP application leads to significant increase in microhardness as compared to untreated control specimens. Laser irradiation has been proposed as an adjunction technique to promote the enamel resistance to acid attack. There are controlled thermal effects in diode lasers (especially in the 810 980 nm) that alter the crystallinity of enamel, and decrease carbonate content, leading to the development of acid-resistant hydroxyapatite [16]. Lessening the replacement of carbonate raises the structural strength of enamel apatite crystals, hence, enhancing valuable opposition to demineralization. But in the current investigation, the laser irradiation alone was not statistically significant as a measure of enhancement of hardness. The observation is consistent with previous investigations which indicate that diode lasers, used alone, might not obtain the desired remineralization and need to be combined with exogenous mineral or peptide sources [17].

Surprisingly enough, a combination of SAP and diode laser produced better outcomes than the two treatments. The reason behind the occurrence of this synergistic effect can be attributed to the laser-induced microstructural changes that increase the permeability of enamel and allow greater penetration of the peptide into the demineralized parts. The further development of a peptide-based scaffold in the laser-modified matrix can also lead to a more efficient hydroxyapatite nucleation and crystal growth. The same synergism was observed when using fluoride or CPP-ACP with laser therapy where enamel hardness and mineral gain were better than monotherapy [18]. These results suggest that a combination of photo- and bio-based methods can be the most effective in maximizing remineralization performance based on complementary remineralization processes: laser-enhanced diffusion and peptide-guided mineral deposition. Clinically, these discoveries help highlight the possibility of combining biomimetic peptides with adjunctive diode laser treatment when it comes to the treatment of incipient carious lesions. Such a two-modal treatment is consistent with the principles of the minimum invasive dentistry which focuses on natural repair and conserving healthy enamel. The increase in enamel hardness of this experiment indicates that this type of combination can be regarded as a possible substitute of early restorative intervention, which might slow down the development of non-cavitated lesions and lead to enhanced long-term stability of enamel. Being an *in vitro* study, the findings should be handled with care. The experimental model does not include biological variables found in the oral cavity, including salivary flow, pH changes, and biofilm of the microbes, which impact on the remineralization processes. Furthermore, the durability of the mineral layer created by laser and peptide was not tested in the long term, as well as its stability against acid challenges in the future. One should consider future in vivo and longitudinal clinical trials to confirm the effectiveness, safety, and the best application regimens of SAP and diode laser combination therapy in real-life situations of the oral cavity.

## Conclusion:

P11-4 self-assembled in significant degrees of enhancement for remineralization of demineralized enamel but did not exhibit improvement with diode laser alone. SAP used with diode laser resulted in the largest recovery in enamel hardness, indicating that the two are synergistic. This can be a promising approach that has the least invasive characteristics in the treatment of early carious lesions.

## Figures and Tables

**Table 1:** Mean surface microhardness (VHN) after remineralization

**Group**	**Treatment**	**Mean ± SD (VHN)**
A	Control (Artificial saliva)	193.6 ± 4.3
B	SAP (P11-4)	263.1 ± 4.7
C	Diode Laser	201.7 ± 7.8
D	SAP + Diode Laser	298.7 ± 6.2

**Table 2:** Multiple comparisons (Tukey's post hoc test)

**Comparison**	**Mean difference (VHN)**	**p-value**
D vs B	35.6	<0.01
D vs C	97	<0.001
B vs A	69.5	<0.01
C vs A	8.1	0.09 (NS)

**Table 3:** Hardness recovery percentage compared to demineralized baseline

**Group**	**% Recovery**
A (Control)	2.10%
B (SAP)	38.60%
C (Laser)	6.30%
D (SAP + Laser)	57.50%
